# Pulmonary lesions in early response assessment in pediatric Hodgkin lymphoma: prevalence and possible implications for initial staging

**DOI:** 10.1007/s00247-024-05859-y

**Published:** 2024-01-31

**Authors:** Dietrich Stoevesandt, Christiane Ludwig, Christine Mauz-Körholz, Dieter Körholz, Dirk Hasenclever, Kathleen McCarten, Jamie E. Flerlage, Lars Kurch, Walter A. Wohlgemuth, Judith Landman-Parker, William H. Wallace, Alexander Fosså, Dirk Vordermark, Jonas Karlén, Michaela Cepelová, Tomasz Klekawka, Andishe Attarbaschi, Andrea Hraskova, Anne Uyttebroeck, Auke Beishuizen, Karin Dieckmann, Thierry Leblanc, Stephen Daw, Jonas Steglich

**Affiliations:** 1 Department of Radiology, University Hospital Halle, Ernst-Grube-Straße 40, 06120 Halle/Salle, Germany; 2 Department of Internal Medicine, University Hospital Halle, Halle/Saale, Germany; 3 Department of Pediatric Hematology and Oncology, University Hospital Giessen-Marburg, Giessen, Germany; 4 Medical Faculty of the Martin-Luther-University of Halle-Wittenberg, Halle/Saale, Germany; 5 Institute of Medical Informatics, Statistics and Epidemiology, University of Leipzig, Leipzig, Germany; 6 Diagnostic Imaging and Pediatrics, Warren Alpert Medical School, Brown University, Providence, RI USA; 7 Pediatric Radiology, IROCRI (Imaging and Radiation Oncology Core - Rhode Island), Lincoln, RI USA; 8 Department of Oncology, St. Jude Children’s Research Hospital, Memphis, TN USA; 9 Department of Nuclear Medicine, University of Leipzig, Leipzig, Germany; 10 Hôpital Armand-Trousseau Sorbonne Université, Paris, France; 11 Department of Paediatric Haematology and Oncology, Royal Hospital for Children and Young People and University of Edinburgh, Edinburgh, UK; 12 Department of Medical Oncology and Radiotherapy, Oslo University Hospital, Oslo, Norway; 13 Department of Radiation Oncology, Medical Faculty of the Martin-Luther-University, Halle (Saale), Germany; 14 Karolinska University Hospital, Astrid Lindgrens Children’s Hospital, Stockholm, Sweden; 15 Department of Pediatric Hematology and Oncology, University Hospital Motol and Second Medical Faculty of Charles University, Prague, Czech Republic; 16 Department of Pediatric Oncology and Hematology, University Children’s Hospital of Krakow, Kraków, Poland; 17 Department of Pediatric Hematology and Oncology, St. Anna Children’s Hospital, Medical University of Vienna, Vienna, Austria and St. Anna Children’s Cancer Research Institute, Vienna, Austria; 18 Department of Pediatric Hematology and Oncology, University Children’s Hospital, Bratislava, Slovakia; 19 Department of Pediatric Hematology and Oncology, University Hospitals Leuven, Louvain, Belgium; 20 Princess Máxima Center for Pediatric Oncology, Utrecht, The Netherlands; 21 Department of Radio-Oncology, Medical University Vienna, Vienna, Austria; 22 Service d’Hématologie Et d’Immunologie Pédiatrique, Hôpital Robert-Debré, Paris, France; 23 Department of Pediatric Hematology and Oncology, University College London Hospitals, London, UK

**Keywords:** Cancer, Computed tomography, Hodgkin lymphoma, Immunosuppression, Lung, Pediatric

## Abstract

**Background:**

Disseminated pulmonary involvement in pediatric Hodgkin lymphoma (pHL) is indicative of Ann Arbor stage IV disease. During staging, it is necessary to assess for coexistence of non-malignant lung lesions due to infection representing background noise to avoid erroneously upstaging with therapy intensification.

**Objective:**

This study attempts to describe new lung lesions detected on interim staging computed tomography (CT) scans after two cycles of vincristine, etoposide, prednisolone, doxorubicin in a prospective clinical trial. Based on the hypothesis that these new lung lesions are not part of the underlying malignancy but are epiphenomena, the aim is to analyze their size, number, and pattern to help distinguish true lung metastases from benign lung lesions on initial staging.

**Materials and methods:**

A retrospective analysis of the EuroNet-PHL-C1 trial re-evaluated the staging and interim lung CT scans of 1,300 pediatric patients with HL. Newly developed lung lesions during chemotherapy were classified according to the current Fleischner glossary of terms for thoracic imaging. Patients with new lung lesions found at early response assessment (ERA) were additionally assessed and compared to response seen in hilar and mediastinal lymph nodes.

**Results:**

Of 1,300 patients at ERA, 119 (9.2%) had new pulmonary lesions not originally detectable at diagnosis. The phenomenon occurred regardless of initial lung involvement or whether a patient relapsed. In the latter group, new lung lesions on ERA regressed by the time of relapse staging. New lung lesions on ERA in patients without relapse were detected in 102 (7.8%) patients. Pulmonary nodules were recorded in 72 (5.5%) patients, the majority (97%) being<10 mm. Consolidations, ground-glass opacities, and parenchymal bands were less common.

**Conclusion:**

New nodules on interim staging are common, mostly measure less than 10 mm in diameter and usually require no further action because they are most likely non-malignant. Since it must be assumed that benign and malignant lung lesions coexist on initial staging, this benign background noise needs to be distinguished from lung metastases to avoid upstaging to stage IV disease. Raising the cut-off size for lung nodules to ≥ 10 mm might achieve the reduction of overtreatment but needs to be further evaluated with survival data. In contrast to the staging criteria of EuroNet-PHL-C1 and C2, our data suggest that the number of lesions present at initial staging may be less important.

## Introduction

Hodgkin lymphoma (HL) is a rare cancer in children under 15 years of age, but it is the most common cancer among adolescents 15–19 years of age [1, 2]. Treatment of HL has become a great success in modern oncology, with survival rates exceeding 90% for patients irrespective of the stage of disease [3–10]. Survivors of classical Hodgkin lymphoma (cHL) are at high risk of secondary malignancies and cardiovascular disease [1, 11–17]. Increased risks persist for decades after treatment and are clearly correlated with the extent of treatment exposure [18].

Disease staging is most frequently based on the Ann Arbor classification [19] with Cotswold modifications [20]. Nowadays, modern staging systems combine anatomic cross-sectional imaging, including computed tomography (CT) and/or magnetic resonance imaging (MRI), with functional positron emission tomography (PET) [21]. The Lugano criteria published in 2014 are the most recent updated consensus of the International Conference on Malignant Lymphomas Imaging Working Group [22, 23].

Disseminated organ involvement of the lung constitutes stage IV disease. Thus, patients are preassigned to a high-risk stage regardless of other prognostic factors. They receive multi-agent chemotherapy with response-adapted radiotherapy restricted to patients with residual disease on interim staging fluorodeoxyglucose (FDG)-PET scans. Compared with their age-matched controls, after lung radiotherapy with 15 Gy to ≤ 25 Gy, patients have an increased risk of lung fibrosis and recurrent pneumonia [18, 24]. The challenge is to maintain high cure rates while reducing adverse therapy-related side effects. Therefore, the role of imaging is critical to differentiate real pulmonary metastases of HL from other causes of lung lesions common within a pediatric population.

The differential diagnosis of pulmonary abnormalities in patients with HL includes a variety of diseases such as infection (e.g., histoplasmosis or tuberculosis), bronchiolitis obliterans, organizing pneumonia, granulomatosis with polyangiitis, drug toxicity, and effects of radiotherapy [25–29]. Given that pediatric Hodgkin lymphoma (pHL) is an immunocompromising disease [30] that facilitates benign lung changes, any given patient may have a mixture of pulmonary entities. Even in the case of histologically proven malignancy, it is impossible for radiologists to reliably distinguish benign from malignant lesions on a single scan [31, 32]. However, this may be possible with hindsight if the behavior of the individual nodule and the patient’s overall outcome are known.

A lung biopsy for definitive staging of lung nodules is challenging due to the low sensitivity and specificity of a lung biopsy and the morbidity of this procedure. Therefore, given the limited ability of imaging to determine whether a single lung lesion is a metastasis or background noise, a pragmatic approach must be adopted. If lung lesions are found without clinical signs of infection and respond to chemotherapy like extrapulmonary, histologically proven manifestations of HL, pulmonary involvement must be assumed.

Since therapeutic decisions are based on initial staging, a retrospective approach looking at how lung lesions respond to therapy to determine their etiology is not feasible. At present, two of the largest cooperative study groups in Europe and North America already take this dilemma into account in their staging definitions of lung involvement: the European Network for Pediatric HL relies entirely on CT-morphologic criteria including size and number of pulmonary lesions, whereas the Children’s Oncology Group from North America also includes metabolic factors (Table 1). The size criteria for all study protocols are not based on published original research data but on consensus.

**Table 1:** Comparison of staging criteria definitions for disseminated Hodgkin lymphoma involvement of the lung [33]. *CT* computed tomography;, *EuroNet-PHL* European Network for Pediatric Hodgkin Lymphoma, *FDG* fluorodeoxyglucose, *PET* positron emission tomography

Imaging modality	Children’s Oncology Group AHOD 1331	EuroNet-PHL-C1	EuroNet-PHL-C2
CT	At least 1 intrapulmonary focus > 1 cm and is PET positive or 3 or more lesions between 0.5 and 1 cm regardless of FDG-PET activity Solitary lung nodules<1 cm in transverse diameter, but FDG avid is considered positive	> 3 small foci between within the whole lung OR At least one intrapulmonary focus of diameter > 10 mm	≥ 3 small foci between 2 mm and 10 mm within the whole lung OR At least 1 intrapulmonary focus of a diameter ≥ 10 mm Note: If all lesions are exclusively in one lung, then only this particular lung is considered involved. However, even if there is just one additional smaller focus found within the other lung, then both lungs are considered involved
PET	≥ 1 cm in greatest transverse diameter by CT, FDG uptake exceeding that of mediastinal blood pool structures. When possible, use ascending aorta or aortic arch for reference mediastinal blood pool <1 cm in greatest transverse diameter by CT, due to partial volume averaging effects, any uptake is considered positive	Not defined	Not defined

Current international collaborative efforts to harmonize the staging evaluation and response criteria in clinical trials for children, adolescents, and young adults with HL are ongoing [33].

As a next step in distinguishing true pulmonary metastases from background noise on initial staging, parameters such as size, number, and morphologic criteria must be considered. One way to solve the problem is to compare the initial imaging with the interim staging during therapy in patients with no recurrence or progression of disease. These new pulmonary lesions might be the result of other causes, such as opportunistic infection due to chemotherapy-induced neutropenia or immunosuppression from HL itself.

Thus, the description of these lesions represents a good approximation of the background noise that complicates the delineation of true pulmonary metastases to initial staging. This distinction is urgently needed to prevent patients from erroneous upstaging to stage IV and for tailoring therapeutic strategies.

This study attempts to:

1. Measure the frequency, size, and pattern of newly occurring lung lesions during chemotherapy on early response assessment.
2. Derive recommendations for initial staging based on the assumption that malignant and benign lung lesions may coexist at this time. Additional new lung lesions arising during chemotherapy are rare and can be assumed to be epiphenomena and therefore benign.

## Materials and methods

This retrospective analysis included patients enrolled in the EuroNet-PHL-C1 trial (EudraCT: 2006–000995-33; Clinicaltrial.gov: NCT00433459). This trial recruited 2,102 pHL patients younger than 18 years with cHL between January 31, 2007, and January 30, 2013. The imaging protocol comprises a CT of the chest for initial staging and for interim staging defined as early response assessment (ERA) on days 29 to 31 of the second cycle of chemotherapy consisting of vincristine, etoposide, prednisolone, doxorubicin (OEPA; 1–5 mg/m^2^ vincristine intravenously capped at 2 mg, on days 1, 8, and 15; 125 mg/m^2^ etoposide intravenously on days 1–5; 60 mg/m^2^ prednisone taken orally on days 1–15; and 40 mg/m^2^ doxorubicin intravenously on days 1 and 15) [9, 10].

Written informed consent was provided by all patients or their guardians. Local and central ethics boards in each country approved the study, which was conducted in accordance with Good Clinical Practice and the Declaration of Helsinki.

**Fig. 1:**
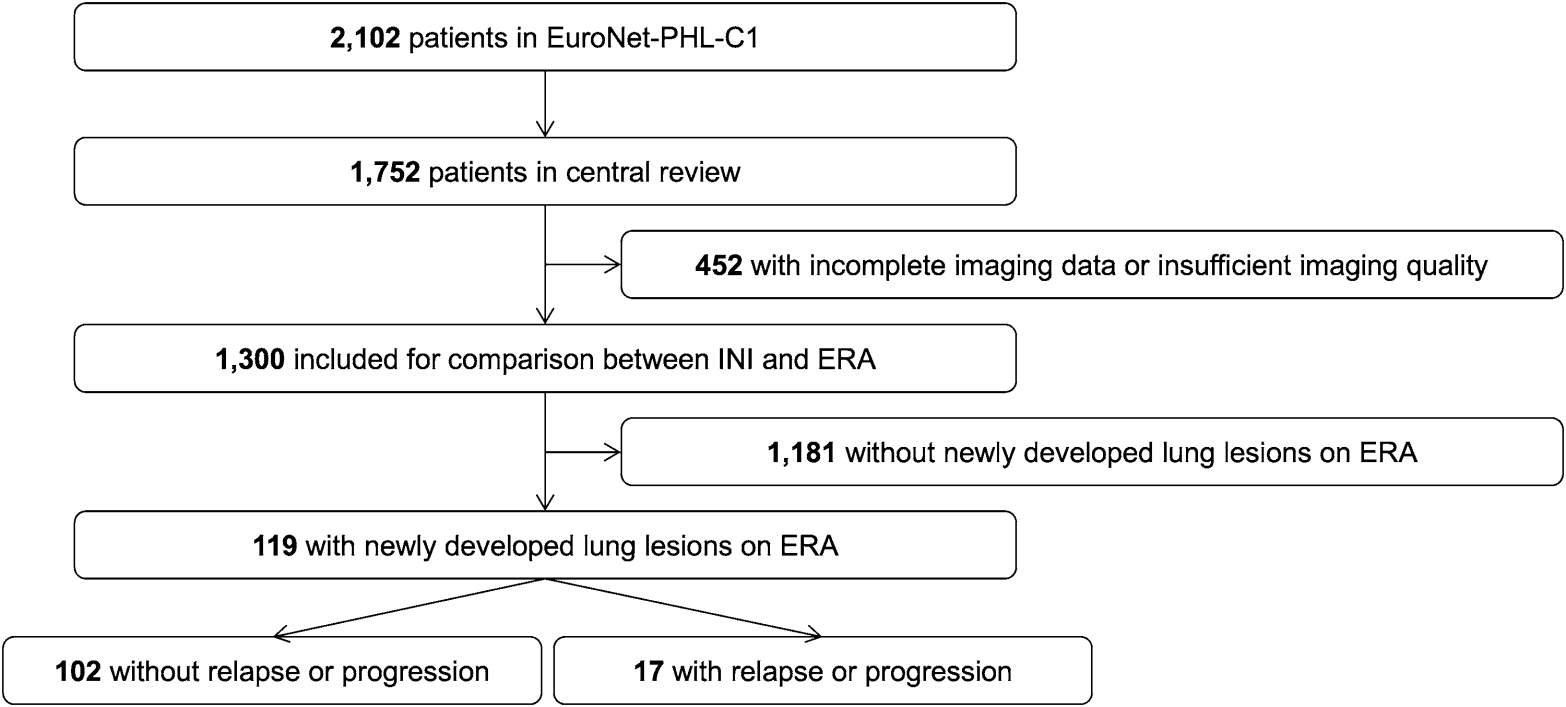
Research design flow chart: selected patients from the EuroNet-PHL-C1 (European Network for Pediatric Hodgkin Lymphoma) trial with newly developed lung lesions appearing between initial staging (INI) and early response assessment (ERA)

Out of 2,102 study participants in the EuroNet-PHL-C1 trial, 1,752 (83.3%) were available for central review. Incomplete or missing images to the central review board and inadequate imaging quality were imaging-related exclusion criteria (Fig. 1). Inadequate imaging quality was defined as significant respiratory or motion artifacts (apparent gaps in lung coverage), slice thickness equal or larger than 10 mm, or low spatial resolution on ultra-low-dose CTs or low-dose CTs acquired only for attenuation correction of PET. This retrospective analysis includes CT examinations of 1,300 patients. Each patient’s initial staging and ERA images were reviewed for new lung lesions that developed during two cycles of OEPA. While PET/CTs are utilized for staging and response assessment [22, 23], this analysis was done without regard to metabolic activity of the lung lesions due to the frequent unreliability and of lack of FDG uptake in small lung nodules (< 1 cm) [34].

To evaluate the presence of pulmonary lesions, three physicians (D.S., radiologist, 15 years of experience; J.S., resident in radiology, 2 years of experience; C.L., specialist in internal medicine and pneumology; 7 years of experience) reviewed CT scans at standard lung window settings (center, -600 HU; width, 1,500 HU). For each examination, the reconstruction parameters of slice thickness, increment, and field of view (FOV) were recorded.

**Fig. 2:**
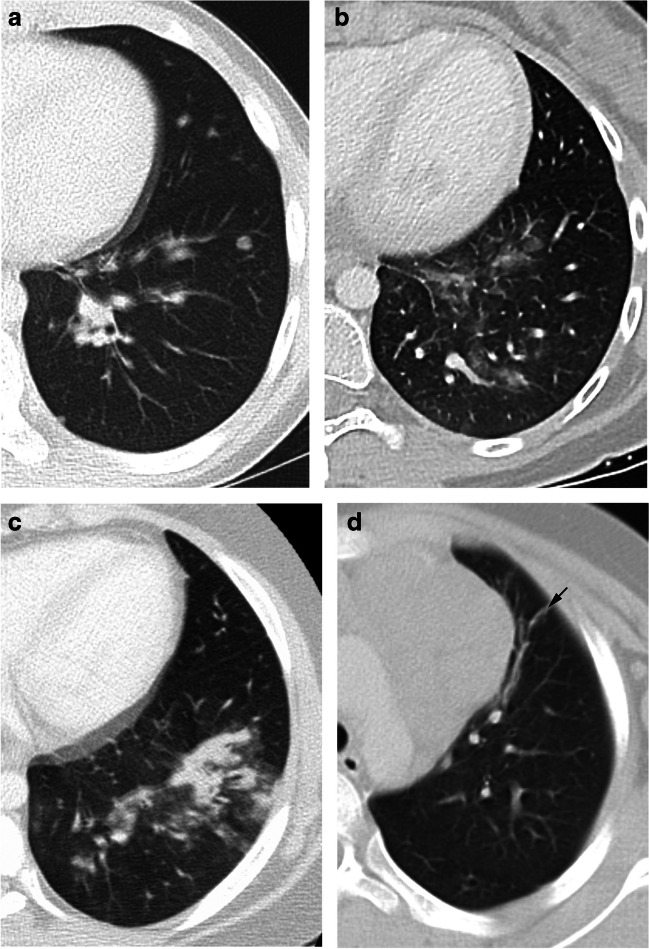
Axial computed tomography scans on lung windows show the different morphological patterns of lung lesions that developed between initial staging and early response assessmen in patients with Hodgkin lymphoma. **a** A nodule in a 14-year-old boy. **b** Ground-glass opacity in a 14-year-old girl. **c** Consolidation in a 12-year-old girl. **d** Parenchymal band (*arrow*) in an 8-year-old girl

A comparison was made between the appearance of abnormalities on ERA and those on initial staging. New pulmonary lesions on ERA imaging were classified as one of the following patterns based upon the currently recommended terms of the Fleischner Society [35] and previously published data on lung involvement in pHL [25, 36] (Fig. 2): (1) nodules, (2) masses, (3) ground-glass opacities, (4) consolidations, (5) parenchymal bands, and (6) perilymphatic distribution.

After defining the pattern of a lung lesion, the following parameters were recorded:

1. Size in millimeters (mm) measured in its largest axial dimension
2. Affected bronchopulmonary segment and lung lobe (right upper lobe (RUL), right middle lobe (RML), right lower lobe (RLL), left upper lobe (LUL), lingula, left lower lobe (LLL))
3. Distance to the visceral pleura in axial slices subdivided into four groups: contact to its surface, peripheral (distance<1 cm), central lobar (distance ≥ 1 cm), hilar
4. Presence of pleural tags, air bronchograms, and cavitations as supplementary characteristics

Documentation of newly developed lung lesions was restricted to ten per patient to limit the impact of a single patient on the pattern-based analysis. If different types of lesions were present, we included them all in a ratio that reflected their frequency in the patient. Data were processed with respect to progression or relapse over a 5-year period. Changes in mediastinal or hilar lymphadenopathy over time were assessed in patients without relapse or progression of disease but with new lung lesions on ERA. The statistical analysis was conducted using R (The R Foundation for Statistical Computing, Version 4.0.2., Vienna, Austria).

## Results

### Patients with new lung lesions on early response assessment

Out of 1,300 patients on ERA, 119 (9.2%) had new pulmonary lesions not detectable on initial staging (Fig. 1).

Imaging was performed by 54 different study sites that used 31 different types of CT scanner from four different manufacturers for ERA CT scans. There was a mean slice thickness of 3.3 mm and a mean increment of 2.9 mm. The mean FOV was 388 mm and images of 35 out of 119 patients were sent to central review with a reconstructed FOV of 500 mm or more. Only 73 (61%) patients received a contrast-enhanced CT scan on ERA. The mean time interval between initial staging and ERA was 67.5 days.

### Patients with new lung lesions on early response assessment and with relapse or progression

Progression while undergoing therapy and/or relapse of disease was documented in 17 of 119 (14%) patients (over a 5-year follow-up period). There was one patient with progressive disease under therapy. Early relapse during the first year after the end of therapy was documented in five patients. Mean time to relapse after the end of therapy was 20 months (range 1–45 months). Relapse staging was not available for central review in six patients, in most because of exceeding the age limit of 18 years and transitioning to adult oncology. Of 11 patients with relapse staging images available for central review, four had lung lesions on relapse. New lung nodules larger than 10 mm on relapse were present in three patients, and one patient developed consolidation.

Concerning their new lung lesions on ERA, nine patients presented with only nodules as new lung lesions on ERA with a size of up to seven mm (range 2 mm to 7 mm). A combination of nodules (size range between 3 mm and 6 mm) and consolidations was present in three patients and five patients had only consolidations. In all 11 patients available to central review, the new ERA lesions had resolved completely on relapse staging.

All 17 patients with relapse or progression of disease were analyzed separately (Fig. 1) from the non-relapse patients to ensure that no malignant lesions were included.

### Patients with new lung lesions on early response assessment but without relapse or progression

The subgroup with new lung lesions on ERA and without relapse or progression consisted of 102 patients with histologically proven cHL. They included 48 female (47%) and 54 male (53%) patients with an age range from 4–17 years (median 14; interquartile range 12.3–16.0).

There were 43 patients with new lung lesions on ERA but without any lung lesions on their initial staging. The other 59 patients already had lesions on initial staging but in different locations: 45 patients presented with only nodules on initial staging, seven had a combination of nodules with other patterns of involvement. Only ground-glass opacities were present in four patients, two patients had only parenchymal bands, and one patient had only a consolidation on initial staging. On average, the nodules already present on initial staging had a diameter of 5.7 mm (min. 1 mm; max. 36 mm). The size reduction of these nodules on ERA averaged 71%.

Of the 59 patients with lung lesions on initial staging, 19 had been diagnosed with disseminated lung involvement according to EuroNet-PHL-C1 staging protocol (Table 1). Seventeen patients in this group had nodules, one in combination with consolidations, and one in combination with parenchymal bands. In two patients, only consolidations were present. Nodules in this group measured 7.2 mm on average (min. 1 mm; max. 36 mm). Nodules larger than 10 mm were present in eight patients. Also in these patients, the average size reduction of the initially present nodules was 82%.

**Fig. 3:**
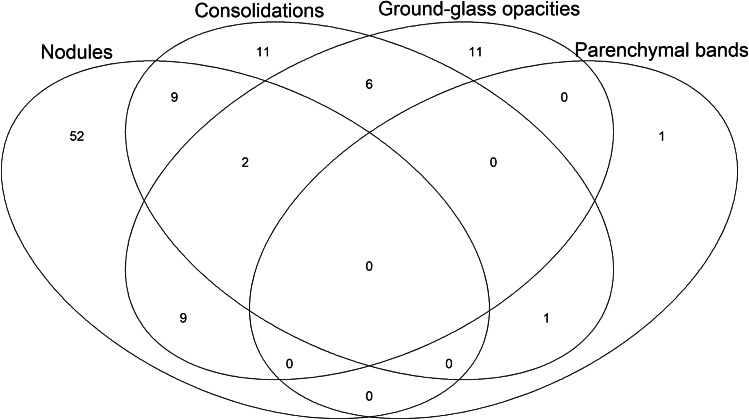
Venn diagram shows the distribution of patients with newly developed pulmonary lesions by morphological pattern (illustrated in Fig. 2) on early response assessment

**Fig. 4:**
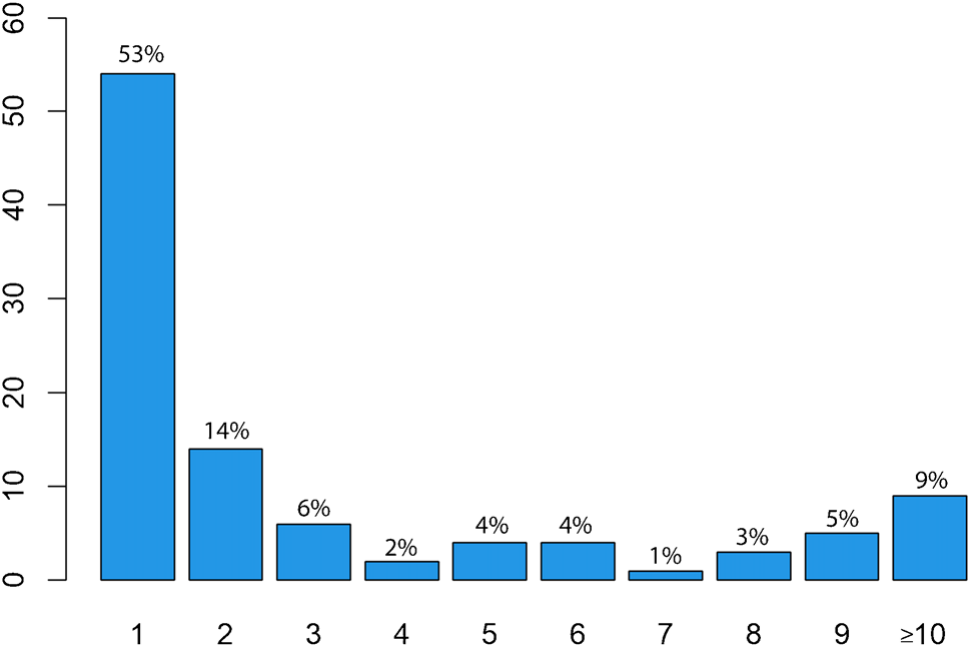
Number of newly developed pulmonary lesions per patient irrespective of their pattern (318 in 102 patients)

Without regard to their pattern, the total number of new pulmonary lesions analyzed was 318. Lesions occurred either bilaterally (30 patients) or restricted to the right (44 patients) or left (28 patients) lung. There were various patterns of new lung lesions on ERA (Fig. 2) as shown in the Venn diagram (Fig. 3). Lung lesions that developed during chemotherapy were either solitary (54 patients) or multiple (48 patients) (Fig. 4). In 52 patients, only new nodules in absence of other lesions were detected and 11 patients developed only ground-glass opacities or consolidations. The most common combination, observed in nine patients, were nodules and ground-glass opacities or nodules and consolidations (Fig. 3). No patient presented with new masses or a perilymphatic distribution.

### Nodules

**Fig. 5:**
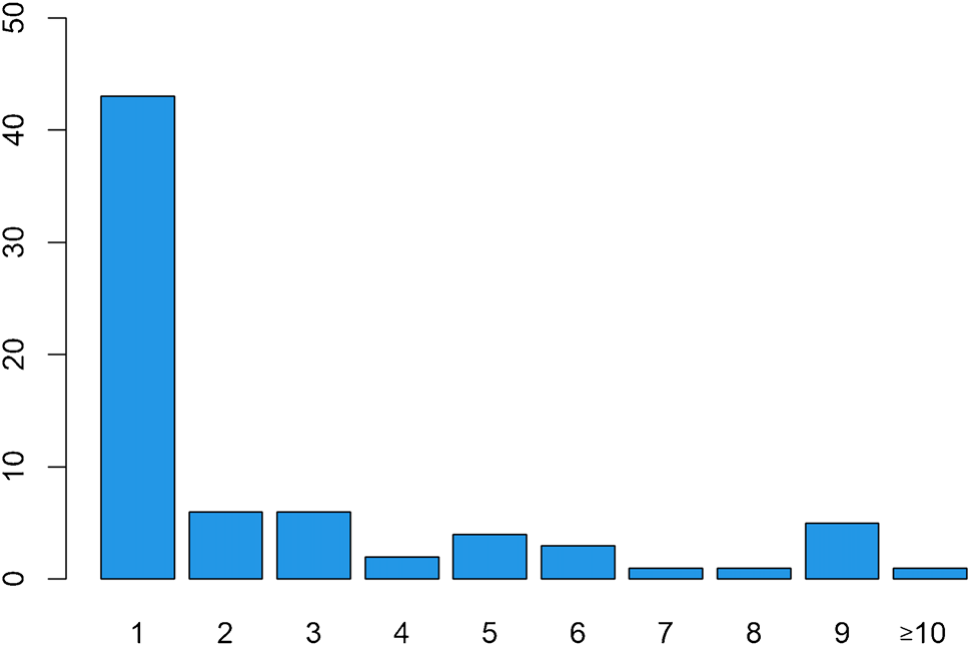
Number of newly developed nodules on early response assessment per patient (189 nodules in 72 patients)

Newly occurring nodules were observed in 72 patients. Of these 72 patients, 43 had only one solitary new nodule, while the others had multiple new nodules (Fig. 5). Exclusively newly emerged nodules in the absence of different patterns were detected in 52 patients. In this subgroup, 39 had only one solitary nodule, and 13 patients showed multiple newly emerged nodules. The total number of analyzed nodules was 189 (out of 318 lesions). There were nodules with sharp (ten), poorly defined (176), and spicular (three) borders. Lobulated nodules or calcifications were not detected. A cavitation was present in three nodules, while pleural tags were present in 7 nodules. There were 118 nodules on the right side (38 RUL, 30 RML, 50 RLL) and 71 nodules on the left side (22 LUL, 5 lingula, 44 LLL) with a predominance of peripheral lung tissue (1 hilar, 65 centrilobular, 102 peripheral, 21 pleural contact) (Table 2). Their diameter ranged from 1 mm to 21 mm (mean 3.68 mm; standard deviation (SD) 2.63 mm). Only seven of 189 nodules (3.7%) were larger than 10 mm.

**Table 2:** Number of newly developed nodules on early response assessment allocated to the bronchopulmonary segments. The included percentages refer to the total of 189 new nodules. *LLL* left lower lobe, *LUL* left upper lobe, *RLL* right lower lobe, *RML* right middle lobe, *RUL* right upper lobe

Right lung *n* (%)			Left lung *n* (%)		
RUL	Apical	15 (7.9%)	LUL	Apicoposterior	15 (7.9%)
	Posterior	6 (3.2%)		Anterior	7 (3.7%)
	Anterior	17 (9.0%)	Lingula	Superior lingula	3 (1.6%)
RML	Lateral	20 (10.6%)		Inferior lingula	2 (1.1%)
	Medial	10 (5.3%)	LLL	Superior	11 (5.8%)
RLL	Superior	17 (9.0%)		Anteromedial	7 (3.7%)
	Medial	0		Lateral	10 (5.3%)
	Anterior	7 (3.7%)		Posterior	16 (8.5%)
	Lateral	13 (6.9%)			
	Posterior	13 (6.9%)			

**Fig. 6:**
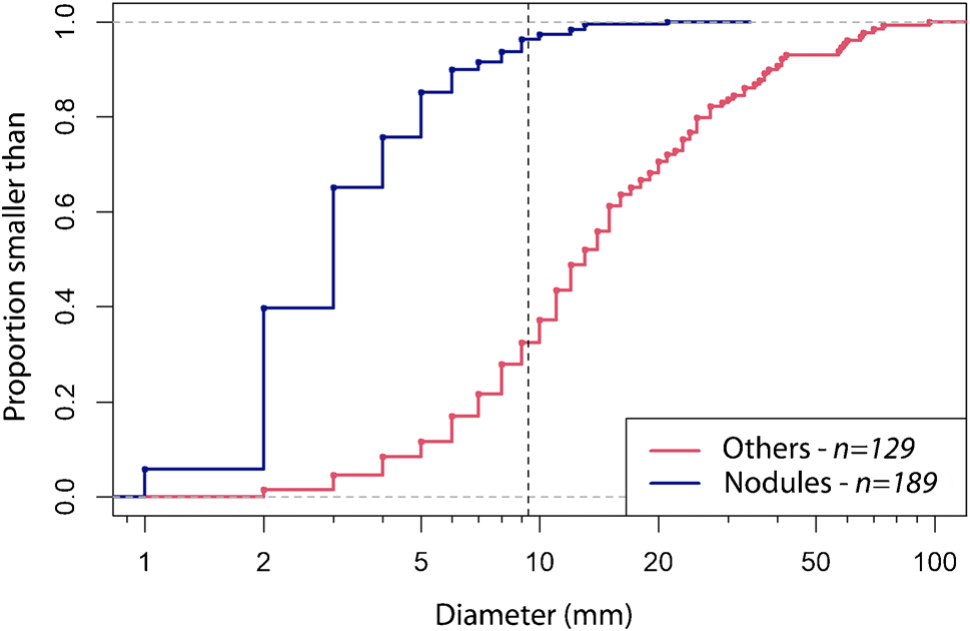
Empirical cumulative distribution function of diameters (mm) of newly developed nodules (*blue*) and other lung lesions (consolidations, ground-glass opacities, and parenchymal bands) (*red*). Most (96.3%) new nodules had a diameter of less than 10 mm

The empirical cumulative distribution function (Fig. 6) shows the cumulative relative frequency of the diameter of newly developed nodules and other lesions. In 96.3% of the nodules that developed, the diameter was smaller than 10 mm, i.e. smaller than the threshold according to the EuroNet staging criteria.

### Ground-glass opacities

Ground-glass opacities appeared in 28 patients. In 11 patients, only ground-glass opacities (in the absence of other lesions) were detected. Of these 11 patients, six had solitary and five multiple ground-glass opacities. The total number of ground-glass opacities was 60. There was a predominance of the right lung with 37 ground-glass opacities (13 RUL, 10 RML, 14 RLL), 23 were on the left side (4 LUL, 2 lingula, 17 LLL). The majority were detected in the centrilobular lung tissue (2 hilar, 29 centrilobular, 18 peripheral, 11 pleural contact).

### Consolidations

**Fig. 7:**
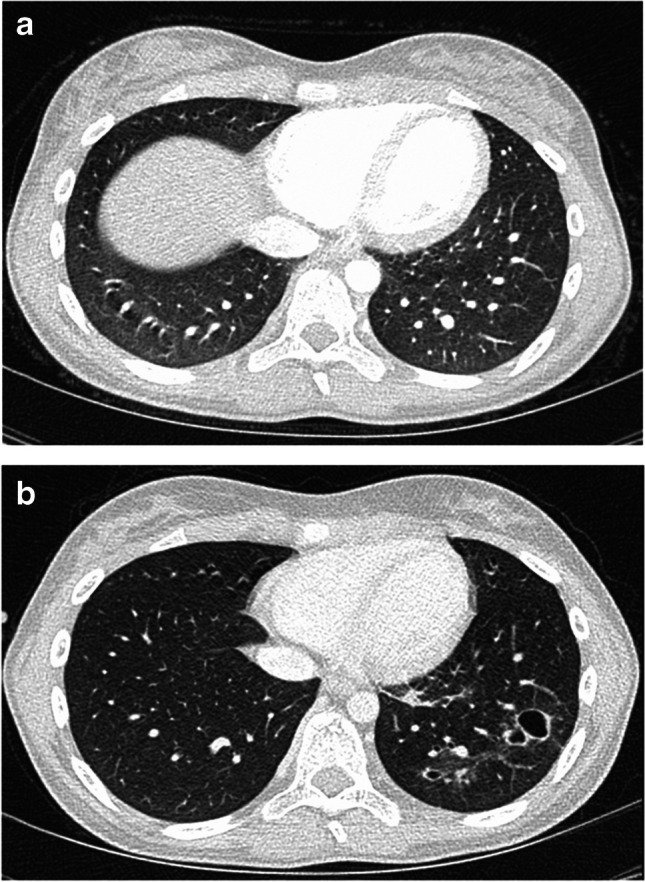
Contrast-enhanced axial computed tomography scans on lung windows in a 13-year-old girl with Hodgkin lymphoma. **a** Initial staging. **b** Early response assessment with newly developed cavitation in the left lower lobe caused by fungal infection facilitated by immunodeficiency

Consolidations were detected in 29 patients. In the absence of any other pattern, they were solitary in eight patients or multiple in three patients. The total number of consolidations was 67. There were 41 consolidations in the right lung (8 RUL, 14 RML, 19 RLL) and 26 in the left lung (0 LUL, 3 lingula, 23 LLL). The majority had contact to the surface of the visceral pleura (2 hilar, 14 centrilobular, 23 peripheral, 28 pleural contact). Air bronchogram was present in 28 consolidations. Cavitating lesions were detected four times. They were found in only two patients (Fig. 7) the same patients with cavitations present in nodules. In a different patient, the consolidation was caused by pulmonary embolism in the RLL artery.

### Parenchymal bands

Parenchymal bands were detected in two patients. In one case, it presented solitary without any other lung lesion and in the other case it occurred combined with consolidations. In the first case, it was situated in the RML and in the second case, it was situated in the LLL. Both of them were located next to the pleura but they had no contact with its surface.

### Dynamics of mediastinal and hilar lymphadenopathy

Involvement of the mediastinum was present in 94 patients (92%), of the right hilum in 57 (56%) and of the left hilum in 46 (45%) patients in the study population on initial staging. Bilateral hilar involvement was present in 37 (36%) patients. On ERA imaging, there was no progression in size of the mediastinal lymphadenopathy. There were four (4%) patients with a size progression in the hilar region (two on the left, one on the right side, one on both sides). In all four patients, there were newly developed ipsilateral consolidations present on ERA.

## Discussion

Disseminated pulmonary involvement of HL is indicative of stage IV disease which requires a more intensive chemotherapy regimen and/or radiotherapy. Correct imaging and appropriate interpretation of abnormalities are even more important for children and adolescents to reduce the risk of secondary malignancies, infertility, and cardiovascular disease and to preserve quality of life [37]. Considering that HL is an immunocompromising disease that facilitates benign lung changes, it is very often unclear if detectable lung lesions are part of the malignancy or non-malignant confounders representing a background noise. The differentiation of these two entities is a prerequisite for an adequate therapy decision.

Biopsy of lung lesions is not feasible in a research context for ethical reasons and because of its low diagnostic yield [38, 39]. Consequently, this paper analyzed new lesions on ERA, because it can be assumed that new lesions developing under chemotherapy that are not associated with relapse or progression can be considered benign in nature and thus most likely represent an approximation of this background noise. There are no published data available on newly developed pulmonary lesions on follow-up images for pHL patients.

There were 119 out of 1,300 (9.2%) patients that presented with new pulmonary lesions on ERA. The phenomenon occurs regardless of whether or not lung involvement was already present at the time of initial staging or even regardless of whether a patient develops relapse. In the latter group, new lung lesions at the time of ERA regressed by the time of relapse staging. Therefore, we assume that new lesions on ERA require no further follow-up imaging because lung progression is extremely rare on ERA.

The majority of newly developed lung lesions were nodules. Consolidations and ground-glass opacities were less common. Many patients presented with a single new nodule, but a minority also developed multiple lesions. Almost all nodules were smaller than 10 mm. No masses were detected. The right lung and the basal lung segments are more likely to be affected which might be explained by lung volume [40] and perfusion [41].

Non-malignant causes of new lung lesions on ERA are diverse. In addition to direct or indirect therapeutic effects, infectious causes may also be considered [42]. No direct pulmonary toxicity has yet been described for the OEPA combination chemotherapy and in accordance with the treatment regimen, there was no radiotherapy between the two points in time of the examinations [9, 10]. It is therefore assumed that lung lesions that developed between initial staging and ERA within approximately 2 months were not caused by direct chemotherapy effects. It is therefore more likely that they are due to opportunistic infections favored by the neutropenia or leukopenia caused by chemotherapy as an indirect effect of OEPA [10, 43] or to the immunosuppression caused by the underlying disease itself [30]. The second mechanism mentioned is a circumstance that is more pronounced in initial staging, as disease activity typically decreases during therapy.

Rosenfield et al. found that even with a known underlying malignancy, approximately 1/3 of biopsied pulmonary nodules are found to be benign. Etiologies included granulomatous disease, infection, inflammatory myofibroblastic lesion, drug reaction, scarring, and intrapulmonary lymph nodes [31]. The Fleischner criteria [44] are not suitable as they relate to incidental pulmonary nodules. Furthermore, they do not comply with patients younger than 35 years, immunocompromised patients, or patients with known cancer.

In our study, no progressive hilar or mediastinal lymphadenopathy was present in any of the patients with new pulmonary nodules; thus, hilar lymphadenopathy does not seem to be a factor to identify infectious causes of new lung nodules.

On ERA, 72 of 1,300 patients (5.5%) had new nodules in the lung mostly<10 mm in size. Maturen et al. found pulmonary involvement in 12% in HL on initial staging with nodules present in 90% of the lung involvement cases [36]. It is morphologically impossible to distinguish lung lesions from lymph nodes in some cases since hilar lymph nodes are present on a lobar, segmental, and subsegmental level [45]. Hilar lymph nodes have been described in up to 60% of the patients in a cohort of 120 children aged 1–17 years who underwent emergency CT after high-energy trauma. They measured up to 9 mm [46].

Pulmonary nodules up to 10 mm in size of benign etiology are therefore a common occurrence in pediatric and adolescent patients with HL.

Most patients had only one newly developed nodule on ERA, but the distribution of patients with two to ten new nodules was relatively homogeneous, suggesting that the number of pulmonary nodules does not appear to be a reliable staging parameter for defining lung involvement. Location and border configuration of the nodules do not seem to be relevant parameters for distinguishing benign from HL related nodules since the benign nodules in our collective are evenly distributed in both lungs and all border configurations occur. The predominance of poorly defined borders is discussed in the limitation section.

In contrast to nodules, newly developed consolidations and ground-glass-opacities present a greater challenge in interpretation because information about antibiotic therapy was not recorded on all staging forms in these patients. In the study by Diederich et al. [25], consolidations on CT before initiation of therapy were detectable in 27% of patients with lung changes in HL, a similar frequency as in our cohort on ERA. Consequently, it is possible for consolidations to be both the result of pulmonary metastatic disease and non-malignant confounders, mainly opportunistic infections. Unfortunately, we were not able to identify a distinguishing morphological feature in our cohort with benign consolidation and ground-glass opacities. Newly developed consolidations in ERA with most likely reactive lymphadenopathy were present in 4 out of 29 patients but hilar or mediastinal lymphadenopathy does not seem to be a reliable parameter for initial staging, as it is common in pHL. As a result, the inclusion of PET, laboratory, and clinical parameters is critical for differentiation.

Although new cavitations on ERA (Fig. 7) were rare, their genesis can be traced from the patient record in both cases: one was a 4-year-old boy who was treated with caspofungin and amphotericin B. After 14 days of antifungal therapy, the lesion fully resolved. A 13-year-old girl was also treated for fungal infection, but no further images were available for central review. In general, fungal infections are not uncommon in an immunocompromised population and should be considered as potential differential diagnosis, but are rare in pHL given the mild degree of immunosuppression compared to chemotherapy for other diseases.

In one patient with a newly developed consolidation, pulmonary artery embolism was present on ERA, so the consolidation should be diagnosed as infarct pneumonia. This cause of consolidations should be considered, especially in tumor patients, although many staging CTs are not feasible due to the contrast agent phase in our collective 39% of the ERA CTs were performed without contrast agent.

Parenchymal bands are usually due to distortion of the lung parenchyma [35]. Newly appearing parenchymal bands on ERA may be due to uneven re-expansion of the lung as the mediastinal tumor conglomerate shrinks. The small number of patients does not allow any further conclusions.

A perilymphatic distribution was not recorded, which could be explained by the decrease in size of the mediastinal mass and thus the removal of the cause of lymphatic congestion.

Although differential diagnostic uncertainty remains with regard to the detectable lesions, an isolated pulmonary progression after two cycles of chemotherapy that spontaneously resolves during further therapy is theoretically possible, but seems very unlikely in HL. CT remains the best option especially since invasive procedures do not provide sufficient sensitivity [31, 38] and are not a suitable alternative from an ethical perspective. As other publications already suggest [32, 47], it is most likely that not every lung lesion detectable on initial staging represents a HL manifestation, but that there is a coexistence of non-malignant and malignant lesions. Consequently, newly developed lesions represent the closest possible approximation to the non-malignant background noise on initial staging. Since the main effort of pediatric HL trials in the past decades was to reduce overtreatment, it may be necessary to rethink the definition of lung lesions on initial staging. Based on our findings in this study and the literature on benign lymph nodes in children [46], we recommend that lung nodules should only be considered disseminated lung involvement if they are larger than 10 mm in diameter. In contrast to the current staging criteria of EuroNet-PHL-C1 and C2, the number of lesions seems to be less important.

In contrast to the current staging protocol for lung nodules, this would allow a proportion of patients with several but small lung lesions to be allocated in lower treatment groups. It would also categorize patients with malignant lung lesions <10 mm into lower treatment groups, but this approach is already common practice established for the definition of involved lymph node regions [33]. Future studies need to closely examine the outcome of these patients. Therefore, nodules <10 mm should still be documented.

In this study, our limited data on cavitations suggests that all cavitations on initial staging should be screened for fungal and other possible infectious causes. The presence of hilar and or mediastinal lymph nodes is not a useful parameter on initial staging since most patients have mediastinal and or hilar involvement.

One major limitation of the approach used in this study to extrapolate phenomena observed at ERA compared to the initial staging is that the degree of immunosuppression at ERA would be more significant compared to that at initial diagnosis, given that chemotherapy was administered placing patients at higher risk for infection. This may result in more subcentimeter nodules due to infectious etiologies, chemotherapy toxicity, etc. In effect, this would overestimate the background prevalence of subcentimeter nodules in pHL patients who are not receiving chemotherapy (i.e. at initial diagnosis).

Other limitations of the study were mainly caused by the high number of participating study centers in this multinational trial, the retrospective study design, and absence of a strict protocol to which participating centers adhered. This resulted in non-standardized protocols being used and therefore in image data-related problems such as low spatial resolution due to an oversized FOV and an excessive slice thickness in a significant number of cases. Especially in small nodules, border configuration can be afflicted by the mean slice thickness of 3.3 mm in our study. The Fleischner Society recommends that for nodule characterization and measurement of small pulmonary nodules, CT scans should be reconstructed with contiguous thin slices (1.5 mm or less in thickness) [44]. Consequently, the number of nodules with poorly defined borders is overestimated in our study. A slice thickness of 1.5 mm or less and a FOV adapted to the patient’s thoracic size should therefore be implemented into the imaging protocols of future trials. Furthermore, it is recommended to use a uniform terminology based on the current Fleischner Society recommendations [35] in order to facilitate the comparison of studies.

A significant proportion of patients recruited in the EuroNet-PHL-C1 trial had to be excluded from the analysis due to unavailability or incompleteness of imaging which reduces the study population and therefore the reliability of the results. The image evaluation and pattern classification of the lesions were done by consensus, which could have influenced the results of the study and its reproducibility.

Another limitation is the missing control group of pHL patients with histologically proven malignant lung lesions. As discussed in the “Introduction” of this paper, lung biopsy is not feasible to distinguish benign from malignant lesions. It has to be assumed that both types of lung lesions coexist in pHL patients on initial staging. Therefore, this study only describes the characteristics of the background noise and is limited in describing discriminating characteristics of benign and malignant lesions.

## Conclusion

Children and adolescents with HL often develop new pulmonary lesions between initial staging and ERA. If there is no progression in lymph node regions on ERA, these lung lesions should not be considered as progression and therefore do not require any further action. While newly developed nodules measured up to 36 mm, the majority of them (96.3%) were smaller than 10 mm. As the favoring conditions for the development of these lesions are also present at the time of initial staging, it must be assumed that benign and malignant lung lesions coexist before starting therapy. To reduce upstaging to stage IV and the associated overtreatment, this background noise needs to be distinguished from true pulmonary metastases. Raising the cut-off size of lung nodules to ≥ 10 mm to define involvement will reduce overtreatment, but this definition needs additional evaluation with outcome data. In contrast to the staging criteria of EuroNet-PHL-C1 and C2, our data suggest that the number of lesions present at initial staging may be less important.

## Data Availability

The datasets generated during and/or analyzed during the current study are available from the corresponding author on reasonable request.
